# Laboratory diagnostics for human *Leishmania* infections: a polymerase chain reaction-focussed review of detection and identification methods

**DOI:** 10.1186/s13071-022-05524-z

**Published:** 2022-11-05

**Authors:** Ineka Gow, Nicholas C. Smith, Damien Stark, John Ellis

**Affiliations:** 1 School of Life Sciences, University of Technology Sydney, Ultimo, NSW 2007 Australia; 2 Department of Microbiology, St Vincent’s Hospital Sydney, Darlinghurst, NSW 2010 Australia

**Keywords:** Leishmaniasis, Diagnostics, Laboratory, Polymerase chain reaction

## Abstract

*Leishmania* infections span a range of clinical syndromes and impact humans from many geographic foci, but primarily the world’s poorest regions. Transmitted by the bite of a female sand fly, *Leishmania* infections are increasing with human movement (due to international travel and war) as well as with shifts in vector habitat (due to climate change). Accurate diagnosis of the 20 or so species of *Leishmania* that infect humans can lead to the successful treatment of infections and, importantly, their prevention through modelling and intervention programs. A multitude of laboratory techniques for the detection of *Leishmania* have been developed over the past few decades, and although many have drawbacks, several of them show promise, particularly molecular methods like polymerase chain reaction. This review provides an overview of the methods available to diagnostic laboratories, from traditional techniques to the now-preferred molecular techniques, with an emphasis on polymerase chain reaction-based detection and typing methods.

**Graphical abstract:**

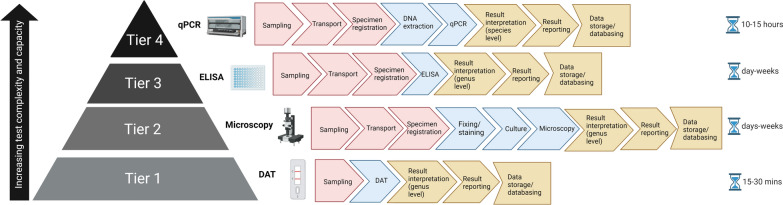

## Background

Among parasitic diseases, leishmaniasis is second only to malaria as a cause of human mortality [1]. Human leishmaniasis is considered a neglected tropical disease (NTD) by the World Health Organization (WHO). It is widespread, occurring in all continents except for Australia and Antarctica, but primarily impacts developing nations in the tropics. However, its endemicity in developed nations is changing due to human migration as a result of war, and expansion in sand fly habitats linked to changes in environmental factors that are often associated with climate change [2–5].

Human leishmaniasis is caused by 20 or more species of the protozoan genus *Leishmania* (Kinetoplastida: Trypanosomatidae). These are often referred to as New World or Old World species based on their geographic localisation either in the Western Hemisphere (specifically, Mexico, Central and South America) or the Eastern Hemisphere (specifically, southern Europe, Africa, the Middle East and parts of Asia), respectively. New World species include *Leishmania infantum*,* Leishmania braziliensis*, *Leishmania guyanensis*, *Leishmania panamensis*, *Leishmania peruviana*, *Leishmania lainsoni*, *Leishmania naiffi*,* Leishmania mexicana* (syn. *Leishmania pifanoi*) and *Leishmania amazonensis* (syn. *Leishmania garnhami*) [6]. Old World species include *Leishmania donovani *(syn*. Leishmania archibaldi*),* Leishmania infantum*, *Leishmania tropica *(syn.* Leishmania killicki*),* Leishmania major* and *Leishmania aethiopica* [6, 7]. Other species that infect humans include *Leishmania shawi*, *Leishmania lindenbergi*, *Leishmania venezuelensis*, *Leishmania martiniquensis*, *Leishmania waltoni* (all in the New World), and *Leishmania arabica* and the newly described *Leishmania orientalis* (in the Old World) [7–9]. It must be acknowledged, and kept in mind, however, that the classification of *Leishmania* is problematic largely due to the use of different genetic markers in evolutionary relationship studies [10, 11]. There have been repeated calls for a consensus classification of the genus, but this has yet to be achieved [6, 12].

**Fig. 1:**
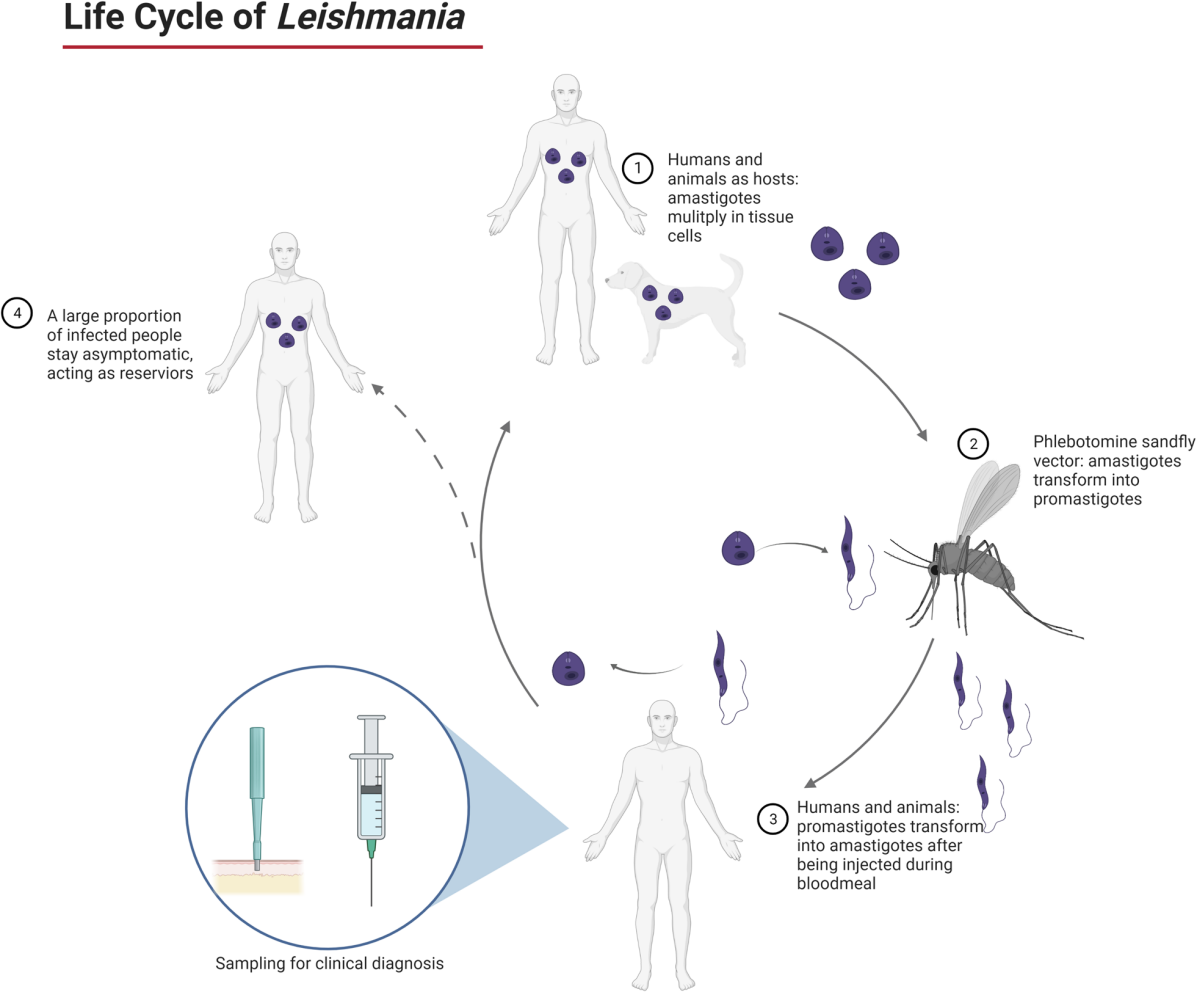
Life cycle of *Leishmania* species. The sand flies inject metacyclic promastigote stages of the parasite as they take their blood meal [13]. These parasites transform into asexually reproducing amastigotes within macrophages and can affect different organs and tissues depending, in part, on the parasite species and the species and immune status of the host. The *Leishmania* species that infect humans are also found in a variety of mammalian reservoir hosts, including canids (particularly domestic dogs), rodents and marsupials [17]

Humans become infected with *Leishmania* through the bite of female sand flies of the genera *Lutzomyia* (in the New World) or *Phlebotomus* (in the Old World) (Fig. 1). Transmission has also been documented through needle sharing, congenital transmission and sexually transmitted infection, albeit rarely [14–16]. Clinical presentation of leishmaniasis consists of two main forms: cutaneous leishmaniasis (CL), which includes manifestations such as mucocutaneous leishmaniasis (MCL), diffuse cutaneous leishmaniasis, disseminated leishmaniasis, leishmaniasis recidivans and post-kala-azar dermal leishmaniasis; and kala-azar or visceral leishmaniasis (VL) (Table 1). Each manifestation may be associated with certain species of *Leishmania*, although there is considerable overlap, exceptions, hybrid species and mixed infections that are recognized [18, 19]. Furthermore, *Leishmania* has been shown to exhibit mosaic aneuploidy, which influences genetic diversity not only within a given species but within a single isolate [20, 21]. The clinical manifestations of *Leishmania* infection may also be affected by the presence of sand fly saliva and the host’s genetic makeup and immune status [22, 23].

**Table 1:** Clinical forms of leishmaniasis

Clinical form	Principal causative agents	Common symptoms and manifestations	Complications, confounding factors and prognosis
Cutaneous leishmaniasis (CL)	*Leishmania infantum, Leishmania donovani, Leishmania tropica, Leishmania major* and *Leishmania aethiopica* (Old World); *L. infantum, Leishmania braziliensis, Leishmania guyanensis, Leishmania panamensis, Leishmania peruviana, Leishmania mexicana* and *Leishmania amazonensis (*New World) [19, 36, 255]	Parasites remain localised, with patients mostly exhibiting non-healing ulcers on exposed regions of the body; a primary lesion (in New World cases) or lesions (in Old World cases) in the form of a red papule occurs 1 week to 3 months after infection, progressing into a larger plaque or nodule weeks after the development of the initial papule; an ulcer with a dark border and crusted base then forms between 1 and 6 months, occasionally paired with painless, rubbery nodules, papules or hardened masses around the site of the ulcer; surrounding lymph nodes can become enlarged and may itch, although pain is mild or absent [79, 161, 183, 254, 256, 271]	Lesions may heal spontaneously at between 1 and 36 months, leaving a discoloured scar with social and psychological consequences for the patient; complete immunity generally occurs; complications include bacterial supra-infection [257]
Mucocutaneous leishmaniasis (MCL)	*L. braziliensis*, *L. panamensis, L. guyanensis* and, occasionally, *L. major* and *L. infantum* (Old World); and *L. amazonensis* (New World) [272, 273]	Characterised by metastatic spread of parasites from the site of the sand fly bite to the upper respiratory tract mucosa, occurring concurrently with cutaneous lesions or up to 5 years after the lesions have healed; initially, reddening and ulceration around the nasal region occurs, followed by destruction of the nasal septum, pharynx and larynx and, rarely, the eyes and genitalia [24]	The disease does not heal spontaneously and healing post-treatment can leave devastating scarring with social and psychological consequences for the patient; complications include malnutrition and pneumonia [22, 260–263]
Kala-azar/visceral leishmaniasis (VL)	*L. donovani* in East Africa and the Indian subcontinent or *L. infantum* in Central and South America, Europe and North Africa [21, 32, 35, 104, 268]	Parasites spread to the liver, spleen and bone marrow from the site of the sand fly bite via macrophages travelling in the blood or lymphatic system; patients present with fever, fatigue, weakness, anorexia and enlargement of the liver and spleen; the incubation period is between 12 and 32 weeks [15, 39, 57, 136]	Complications include co-infections with human immunodeficiency virus, bacterial pneumonia, tuberculosis, dysentery; fatal if left untreated, often due to severe anaemia [258, 259]
Diffuse cutaneous leishmaniasis	*L. aethiopica*, *L. infantum* (Old World);* L. mexicana, L*. *amazonensis* (New World) [264, 270]	Presents as mixed lesions and plaques affecting limbs, buttocks and face due to an anergic response, usually in immunocompromised patients, producing non-ulcerative nodules that become chronic with a high parasite load [77, 264, 265]	A rare complication of cutaneous leishmaniasis; visually, very hard to distinguish from leprosy [264, 271]
Disseminated leishmaniasis	*L. braziliensis, L. mexicana* (New World only) [266]	Presents as mixed-type lesions on multiple sites of the body, often including the mucosal regions, and a low parasite load in skin [266]	A rare complication of cutaneous leishmaniasis [266]
Leishmaniasis recidivans	*L. braziliensis* (New World); *L. tropica* (Old World) [267]	Characterised by red papules arising from within the borders of healed cutaneous lesions and slowly progressing into chronic recurring nodules; may be a recurrence of a prior infection, occurring after months/years of dormancy; often affecting the face [18, 28, 267]	A rare complication of cutaneous leishmaniasis [24]
Post-kala-azar dermal leishmaniasis	*L. donovani, L. infantum* [269, 274, 275]	Cases are rare and location-specific: in Africa, symptoms include a rash of papules on the face, ears and forearms, which may heal spontaneously after a few months; in India, small macules that progress into large irregular patches on chest, back, neck and both thighs and arms, before developing into soft, painless, non-ulcerating nodules on the face, ears, trunk and genitals or, sometimes, on the hands and feet [17]	A rare complication of VL; visually, very hard to distinguish from leprosy and confirmation by microscopy may be problematic due to the low parasite load associated with the condition [24]

Despite being listed as a NTD in 2007, and being the subject of concerted global efforts for its control, the incidence of leishmaniasis remains significant, and the risk to vulnerable, mostly poor, populations remains lamentably high [24, 25]. In 2018, over 200,000 new cases were reported to the WHO, and it is now estimated that there are 1 billion people at risk of contracting *Leishmania* [26, 27]. These figures are probably underestimates, as *Leishmania* is not always a notifiable disease and treatment is not always sought due to financial or geographic constraints [28, 29]. Mortality rates are also under-reported, being confined mainly to official hospital deaths. VL has case fatality rates of between 1.5%, in Bangladesh, to 20%, in South Sudan; based on a global case fatality rate of 10%, these figures represent approximately 20,000–40,000 deaths per year [30]. In recent years (2006–2016), morbidity related to leishmaniasis, in terms of disability-adjusted life years, has risen by 12.5% for CL/MCL but decreased by 61.1% for VL [31]. Other burdens exist, such as psychological morbidity arising from the social stigma surrounding the disfiguring lesions or scarring caused by CL and MCL [17].

## The importance of the detection and identification of *Leishmania*

Worldwide control of leishmaniasis is believed to be achievable, but it does present a multifactorial problem because transmission of *Leishmania* species takes place in a complex biological interplay involving a human host, a diversity of parasite species, sand fly vectors, and animal reservoirs of infection, and is affected by a number of factors, including climate change, deforestation and war. Asymptomatic infections (and paucisymptomatic infections) add a further challenge, as undiagnosed patients become unobserved reservoirs of the disease, contributing to further transmission and maintenance of leishmaniasis foci [24, 32, 33]. Mild cutaneous infection, such as *L. major* CL infection, often goes undiagnosed and untreated, especially in resource-limited settings, which increases the risk of spread to the community [34]. Asymptomatic infections have been identified in several screening studies, including those of blood donors, and some studies have found these to be more common than symptomatic infection [35–38]. This was quantified in an epidemiological survey of peripheral blood by using real-time polymerase chain reaction (PCR), where the authors were able to determine a threshold of five parasitic genomes per millilitre of blood at which asymptomatic disease progressed to symptomatic disease [39].

**Fig. 2:**
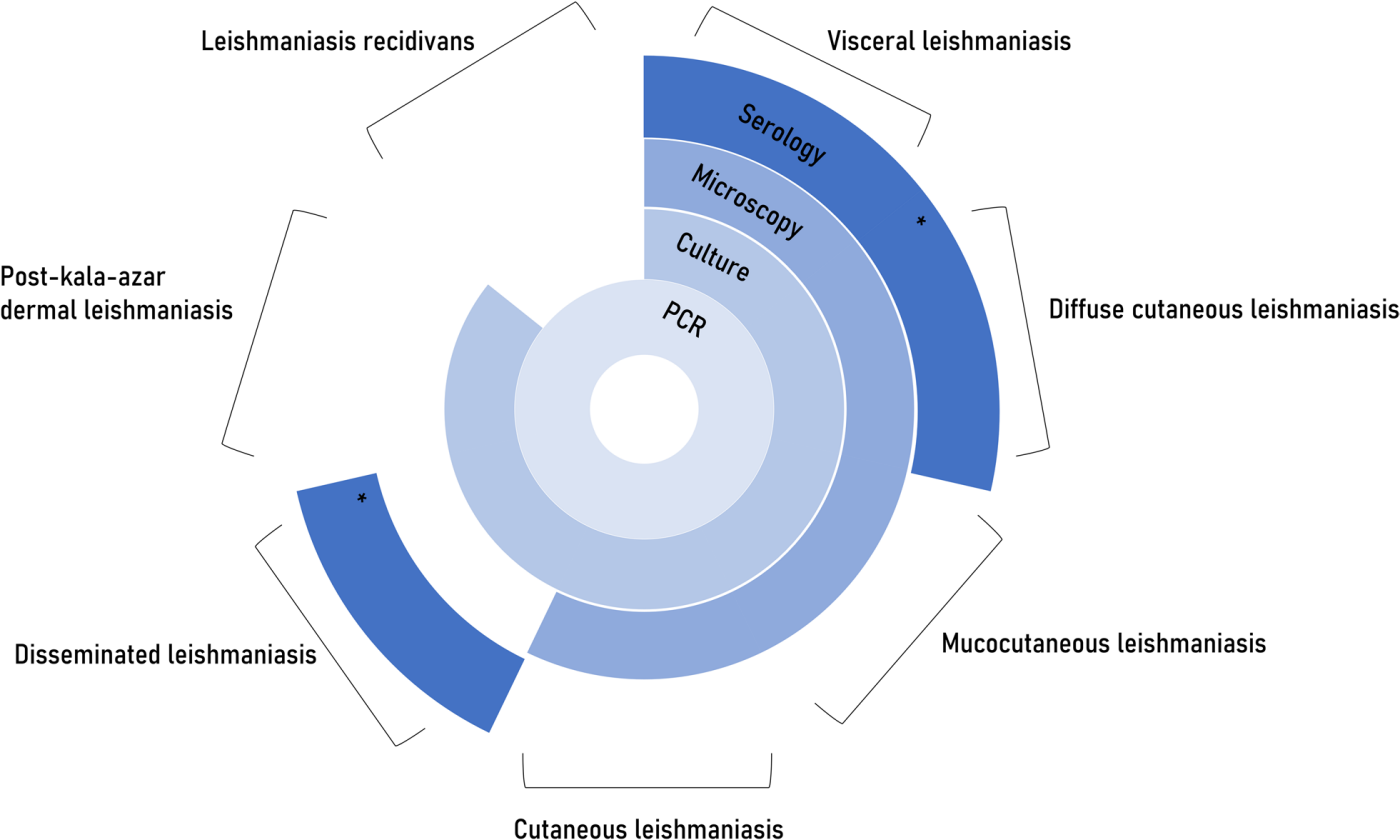
Recommended laboratory-based diagnostic techniques based on clinical presentation. For diffuse cutaneous leishmaniasis, disseminated leishmaniasis and leishmaniasis recidivans, where recommendations are not available from a public health organisation or reference laboratory, guidance is taken from relevant studies [43–50]. The asterisk indicates the rK39 rapid immunochromatographic test kit only.* PCR* Polymerase chain reaction

One particularly confounding factor for any leishmaniasis control program is that *Leishmania* infection induces a broad spectrum of disease states and the clinical presentation cannot be linked to individual species with certainty; moreover, geographic foci may harbor multiple species. Thus, diagnosis on clinical grounds alone, through physical examination and interrogation of patient travel history, is not sufficient for complete case assessment. Figure 2 shows the recommended diagnostic techniques for the various clinical forms of leishmaniasis given by the WHO, the Centers for Disease Control and Prevention, the Walter Reed Army Institute of Research, the National Reference Centre for Parasitology (Canada) guidelines, and relevant studies. For clinical case management, species-level data provide important information for educated prognosis and therapeutic decision-making. For instance, species-led treatment was found to be imperative for cutaneous leishmaniasis in Peru, as tolerance and susceptibility to antimony was dependent upon the infecting species [40]. At a national and global level, the epidemiological monitoring of individual species and their prevalence and transmission patterns guide public health responses, such as the VL elimination program launched in 2005 in India, Nepal and Bangladesh [41]. Moreover, tracking the presence of exotic species, e.g. *L. tropica* discovered in a returned traveller to Mexico, allows local health authorities to enact sanitary measures (such as indoor residual spraying) [42]. Consequently, a globally applicable technique that captures both genus- and species-level data has been suggested, to mitigate assay design complications, such as intraspecies heterogeneity and gene target copy number variations, and to detect asymptomatic and multiple-organism infections (including co-infection with multiple *Leishmania* species) [6]. Seven criteria have been proposed for species-typing tools: discrimination of species, global applicability, sensitivity, specificity, standardisation, applicability for particular settings, and validation [6].

Additionally, test availability and timeliness affect opportune treatment. Shortages of diagnostic materials and a long period of time needed to deliver diagnostic results impede patient outcomes [51, 52]. These limitations result in a lack of useful information for medical decision-making. Furthermore, many current diagnostic tests do not have the resolving power required to provide molecular epidemiological data for public health policy makers. Accurate and qualitative detection and identification of a *Leishmania* infection are, therefore, key to the diagnosis of leishmaniasis, and should be at the heart of any successful control program. This can lead to the achievement of early and improved treatment regimens, implementation of control measures leading to better patient outcomes, and a reduction of sustained reservoirs in the transmission cycle [53]. Additionally, optimised diagnostic tools would be important additions to the One Health approach for the control of leishmaniasis [54]. The comparative strengths and weaknesses of existing diagnostic approaches, with a focus on the widely used and robust PCR-based methods that address many of the requirements for an optimal diagnostic test, are reviewed below.

## Methods for the detection and diagnosis of leishmaniasis

### Conventional detection methods

Detection via microscopy, histology, culture and serology, and other methods, are commonly utilised by laboratories globally, and especially in endemic, resource-poor nations [55].

For microscopic detection of parasites, direct aspirate smears are used, often with staining; amastigotes appear round in shape and 2–4 μm in diameter, and cultured promastigotes range between 15 and 25 μm in length and are ellipsoid to slender in shape [56–58]. Staining methods help to clarify the cells and Giemsa and Leishman stains (both derivatives of Romanowsky stain) are the most widely used for this [59]. Upon staining, *Leishmania* amastigotes are generally observed within macrophages and have a pale blue cytoplasm, red nucleus and adjacent purple-pink-stained kinetoplasts [60, 61]. Parasitic load may be estimated using the modified Ridley’s parasitic index (Table 2), which quantifies the number of amastigotes [62]. The sensitivity of detection varies (54.0–96.4%), and specificity as low as 46.0% has been reported, depending upon the primary sample taken, the quality of the reagent used for staining and technical expertise [43, 63–68]. The stage of infection can also greatly affect sensitivity; in CL, amastigote levels decrease as infection progresses, including in MCL infection, whereas in VL, parasitic load increases as infection becomes chronic [69–71]. Generally, in VL, the highest sensitivity when using microscopy is for more invasive specimens, such as those seen in splenic aspirates [70, 72, 73]. Microscopy is of use at the genus level, but cannot be used for species differentiation as all species of *Leishmania* are morphologically very similar [74]. Recently, machine learning has been incorporated into microscopical examination for leishmaniasis, with sensitivity and specificity of 83% and 35%, respectively, although efficacy and speed are dependent on image quality and the particular algorithm employed [75].

**Table 2:** Modified Ridley’s parasitic index [76]

Parasitic index	Number of amastigotes per standard section
1+	≥ 1
2+	≥ 10
3+	≥ 100
4+	≥ 1000
5+	≥ 10,000
6+	≥ 100,000

Histological examination for CL cases uses 4- to 5-μm tissue sections, stained with Giemsa or haematoxylin and eosin stains, and fixation to reveal histiocytes containing intracellular amastigotes, often near the epidermis [77]. The marquee sign, where organisms are located around the periphery of the dermal macrophage, is regarded as a typical characteristic of leishmaniasis [78]. Histological examination does, however, depend upon the disease stage, since the number of amastigotes decreases as CL progresses until they are undetectable [62]. Indeed, it is considered the least sensitive diagnostic method, with sensitivities of between 42.0% and 70.0%, although 100% specificity has been reported [79–81]. Furthermore, *Leishmania* cells may be mistakenly identified in histological sections as* Toxoplasma gondii*,* Mycobacterium leprae*, fungi, including* Histoplasma*, or even artifacts, and differential diagnosis requires alternative stains [81–84]. Histological examination is less used in VL, where clusters of histiocytes, amastigote presence or morphological changes may be observed [85]. Amastigotes are often unevenly distributed, as sections are of varying thickness, which results in lengthy analysis [86].

Culture of *Leishmania* promastigotes is a useful tool for increasing the sensitivity of downstream detection and identification by microscopy or molecular applications [87]. A variety of semi-solid, liquid or biphasic media are used to culture promastigotes, including sloppy Evans, Novy-MacNeil-Nicole (the reference medium for isolation), Tobie’s, Schneider’s *Drosophila* medium, Senekjie’s, Medium 199, RPMI 1640, Grace’s insect medium, brain–heart infusion medium, blood agar (including rabbit blood) and chocolate agar [88–90]. These media generate growth in differing ways; of note, Tobie’s medium encourages the transformation of amastigotes to promastigotes, and cell density is increased on Grace’s medium [73]. Novel culture methods include the microcapillary culture method, which concentrates the sample in capillary tubes, or liquid (single-phase) media, used to create the microaerophilic conditions that are optimal for amastigote transformation into promastigotes [91]. One study found an improvement from 69.2% sensitivity with traditional culture methods to 92.3% sensitivity with the newer microcapillary method, with a minimal change in specificity (98.9% vs 97.8%, respectively) [92]. It can take days to weeks to produce a result by culture methods, which are also expensive and labour-intensive to set up [86]. Furthermore, the distribution, transport and storage of cultures and culture material, including antibiotics used to prevent contamination from other microorganisms, make it an impractical method in many clinical settings [93, 94].

Serological methods test for *Leishmania* by detecting antigens or antileishmanial antibodies in the blood or, sometimes, urine or saliva. Antibody detection is primarily used in cases of VL rather than for CL, as the humoral response to the latter is poor [95]. Many antigens have been assessed for antibody detection, and recombinant antigens are preferred over natural antigens, as the latter often cause problems such as cross-reactivity and resultant false-positive results [61, 96]. Some immunoassays, such as enzyme-linked immunosorbent assay (ELISA) and western blot, require relatively sophisticated and expensive equipment and materials, which renders them less useful in endemic regions in poorer countries, despite their good sensitivities and robustness [97, 98]. However, ELISA is widely used as a serological method in countries where leishmaniasis is endemic or non-endemic, and it can provide detailed information on antibody responses [99, 100]. The sensitivity of ELISA depends on the antigen used to capture a specific antibody, with the commonly used crude soluble antigen (CSA), for example, providing sensitivities of between 80 and 100%. However, cross-reactions with trypanosomiasis, tuberculosis and toxoplasmosis have been observed with this method [101]. Flow cytometry for serological *Leishmania* detection is a recent development and can quantify antibodies rapidly with lower sample input volumes than other serological tests [102]. Despite the range of tests available and the ability of some of these tests to be used in the field, serological assays share the same limitations. The antigen load may not be sufficient in early infection for detection; conversely, because antigen-specific antibodies persist long after cure, active relapsed disease cannot always be discerned [61, 103]. Furthermore, these tests are less accurate for immunocompromised patients, and cross-reactivity is reported with other diseases endemic to *Leishmania*-affected areas, including Chagas disease [53, 88, 104–108]. Simplified assays have been introduced, such as the rK39 immunochromatographic assay (ICT), the direct agglutination test, the indirect fluorescent antibody test and latex agglutination testing [109]. They include the Food and Drug Administration-approved ICTs, CL Detect, based on the peroxiredoxin antigens, and Kalazar Detect, based on a 39 amino acid repeat recombinant leishmanial antigen, rK39. Although the rk39 ICT is widely used, its sensitivity has been found to be higher in some regions, with studies carried out in Southeast Asian countries and the Indian subcontinent documenting higher sensitivities than those undertaken in East Africa and Brazil [110–112]. Furthermore, because antibodies are present in asymptomatic individuals and remain present for several years after cure, antibody tests must be used in conjunction with strictly standardised clinical case definition (i.e., more than 2 weeks of fever, weight loss and splenomegaly) for VL diagnosis [24]. These limitations must be considered in a modern diagnostic setting, hence, serological methods are being reassessed as principal diagnostic options [86, 113].

Other, lesser-used, methods include the leishmanin (or Montenegro) skin test. In CL, this test can detect past and active cases with high sensitivity, whereas in active VL, the test shows negative as patients are anergic, so it is used in screening studies for an indication of past exposure only [57, 114]. It involves intradermal injection of antigen, whereby induration of an area of the skin of 5 mm or greater is considered a positive test result; sensitivity and specificity with this cut-off point have been reported as 97.4% and 93.9%, respectively [15, 115, 116]. When promastigote levels are too low for culture growth, xenodiagnosis can be used to detect *Leishmania*, through inoculation of the footpad of a hamster with a test sample; however, this approach is time-consuming and involves euthanasia of the hamster [56, 117, 118].

### DNA-based detection methods

In DNA-based detection of *Leishmania* a multitude of genomic targets are used which vary in sensitivity and are influenced largely by the target’s copy number in the genome of the organism. Table 3 summarises the targets that have been investigated for diagnostics. The design of molecular diagnostics is complex, however, and aside from the copy number of a chosen target, which is selected to increase the sensitivity of a given assay (for instance, the 18S rRNA gene), other attributes are sought in molecular assay design [119, 120]. For detection to the subgenus, species complex or species level, selection of gene targets exhibiting increased divergence may necessitate a loss of assay sensitivity by using a target with fewer copy numbers per cell (such as the mini-exon gene) [121]. For instance, polymorphisms, copy number variation and high copy number are attractive features of the kinetoplast DNA (kDNA) minicircle, whereas single copy genes, despite their decreased sensitivity, may be chosen for their stability or to normalise minicircle copy numbers [122, 123]. Moreover, detection of multiple species distinctly and concurrently requires multiplex PCR assays, and designing a single PCR cycling protocol to suit each primer pair can present assay constraints. The use of the novel bisulphite-conversion technique can mitigate such limitations [124].

**Table 3:** DNA targets investigated for the detection of *Leishmania* species in DNA-based methods

Gene	Location	Number of copies^a^	References
6-Phosphogluconate dehydrogenase (*6pgd*)	Chromosomal DNA	S	[276]
18S rRNA	Chromosomal DNA	M (200)	[124]
7SL RNA	Chromosomal DNA	M	[277]
A2 (5′A2 rel, 3′A2 rel, internal A2 rel)	Chromosomal DNA	S (CL)/M (VL)	[278]
Amino acid permease 3 (AAP3)	Chromosomal DNA	M	[279]
Calmodium intergenic spacer	Chromosomal DNA	M	[280]
Casein kinase	Chromosomal DNA	S	[281]
Catalytic subunit of DNA polymerase α (POLA)	Chromosomal DNA	S	[282]
Chitinase	Chromosomal DNA	S	[283]
Cysteine protease A (*cpA*)	Chromosomal DNA	S	[230, 284]
Cysteine protease B (*cpB*)	Chromosomal DNA	M	[230, 285]
Elongation factor-1α (EF-1α)	Chromosomal DNA	S	[286]
Glucose-6-phosphate dehydrogenase (*g6pd*)	Chromosomal DNA	S	[41]
Glucose phosphate isomerase (*gpi*)	Chromosomal DNA	S	[287]
Glyceraldehyde-3-phosphate dehydrogenase (GADPH)	Chromosomal DNA	M	[288]
Heat-shock proteins (HSP)—*hsp10, hsp40, hsp60, hsp70* (M 1–15), *hsp83, hsp90, hsp100, hsp110*; and small HSPs—*hsp20* (S) and *hsp23* (S)	Chromosomal DNA	S/M	[289]
Histones: H2A, H2B, H3, and H4) and linker histones (H1 and H5)	Chromosomal DNA	M	[290]
Hydrophilic acylated surface protein A and B (HASPA/HASPB)	Chromosomal DNA	S	[291]
Intergenic spacer (*igs*) rRNA	Chromosomal DNA	M	[66]
Internal transcribed spacer 1 (ITS1)	Chromosomal DNA	M	[232, 292]
Internal transcribed spacer 2 (ITS2)	Chromosomal DNA	M (50–350)	[232, 293]
Iso-citrate dehydrogenase (*icd*)	Chromosomal DNA	S	[41]
Large subunit rRNA (5.8S, 5S and 28S rRNA)	Chromosomal DNA	M	[232]
*Leishmania*-activated C-kinase antigen (LACK) gene	Chromosomal DNA	M (2)	[235]
Lipophosphoglycans (*lpg*)	Chromosomal DNA	S	[294]
Macrophage migration inhibitory factor (*mif*)	Chromosomal DNA	S	[232, 292, 295]
Major surface protease (*msp*)/glycoprotein 63 (*gp63*)/leishmanolysin	Chromosomal DNA	M (7–70)	[232, 293, 296]
Mannose phosphate isomerase (*mpi*)	Chromosomal DNA	S	[133]
Meta1/2	Chromosomal DNA	S/ M (3)	[297]
Mini-exon [or spliced leader (SL) RNA)	Chromosomal DNA	M (50–650)	[298]
Mitogen-activated protein kinase (MAPK): MAPK2, MAPK3, MAPK4, MAPK5 and MAPK7	Chromosomal DNA	S	[218, 296]
MSP associated gene (*mag*)	Chromosomal DNA	M (18)	[282]
*N*-acetylglucosamine-1-phosphate transferase (NAGT)	Chromosomal DNA	S	[289, 299]
Pteridine reductase 1 (PTR1)	Chromosomal DNA	S	[300, 301]
Repetitive nuclear DNA sequences (REPL)	Chromosomal DNA	M	[282, 302]
RNA polymerase II largest subunit (RPOIILS)	Chromosomal DNA	S	[299, 303]
SIDER repeat	Chromosomal DNA	M	[282]
Small hydrophilic endoplasmic reticulum-associated protein (SHERPs)	Chromosomal DNA	S	[304]
Splice leader associated retrotransposons (SLACS)	Chromosomal DNA	M	[282]
Telomeric sequences	Chromosomal DNA	M	[305]
Tryparedoxin peroxidase	Chromosomal DNA	M (3)	[306]
Tubulins: alpha, beta, gamma, zeta and epsilon tubulin	Chromosomal DNA	M	[218, 282]
Triose-phosphate isomerase (TIM)	Chromosomal DNA	M (2)	[278, 307]
Topo isomerase II	Chromosomal DNA	S	[286, 308]
12S, 9S	Non-chromosomal DNA	M	[295, 309]
Conserved minicircle region (CSB-I, CSB-II and CSB-III)	Non-chromosomal DNA	M (10,000)	[162, 276]
Cytochrome oxidase (CO) I, II and III	Non-chromosomal DNA	M	[290, 310]
Cytochrome b (*cytb*)	Non-chromosomal DNA	M	[282, 311]
Maxicircle divergent region (DR)	Non-chromosomal DNA	M	[133, 312]
Variable minicircle region	Non-chromosomal DNA	M (10,000)	[303, 313]

*rRNA* Ribosomal RNA

^a^Single copy (*S*) or multi-copy (*M*) (approximate number, if available)

DNA-based methods represent a new era in clinical *Leishmania* diagnostics, where the limitations of previous detection methods have been overcome in terms of sensitivity, specificity, rapidity, ease of use and access in endemic settings. Similarly, the prices of molecular methods are decreasing globally; Table 4 illustrates the varying costs associated with *Leishmania* diagnostics in low income and upper-middle income nations [125–128]. The costs associated with molecular methods are comparable to those of other detection methods; however, the methodologies used to collect the costing data varied between studies. Some studies compiled comprehensive costings, including that of the healthcare setting that the test may be performed in, to the basic supplier cost of the kit [125, 126]. Despite their differences, these cost analyses were performed at similar times, and their costing conclusions are similar. These advances have also provided new data on frequencies of asymptomatic carriage, and have been used in the monitoring of specific geographic disease burden and to measure the outcomes of intervention programs [109]. Techniques such as real-time PCR can give more information on parasite load and responsiveness to therapy than other methods [122]. It can be used to identify asymptomatic patients who carry the infection, or infected patients before the onset of symptoms, which is important for the development of control measures and for blood donor monitoring [129, 130].

**Table 4:** Comparison of costs associated with *Leishmania* diagnostics

Country	World Bank income classification	Presentation	Costs (in parentheses)	Associated costs	Year for which costs were determined	References
Colombia	Upper-middle income	MCL	Biopsy + culture + stains + IFA + MST (USD 172.40); biopsy + culture + stains + IFA (USD 162.57); biopsy + stains + IFA (USD 128.91); PCR-mini-exon (USD 128.77); PCR-kDNA (USD 128.77)	Direct	2015	[128]
Afghanistan	Low income	MCL, CL	Microscopy (USD 53.79); RDT (USD 53.91); LAMP (USD 60.18)	Direct and indirect	2016	[127]
Iran	Upper-middle income	CL	PCR–RFLP (USD 5.72); PCR sequencing (USD 11.20); PCR -HRM (USD 4.46)	Basic kit tariff	2015	[126]
Brazil	Upper-middle income	VL	IT LEISH (USD 6.57); DAT-LPC (USD 4.92); Kalazar Detect (USD 7.45); IFAT (USD 11.39); bone marrow aspirate (ambulatory setting) (USD 27.10); PCR USD 32.72	Direct	2016	[125]

*IFA* Indirect immunofluorescence assay,* MST* Montenegro skin test,* RDT* CL Detect Rapid Test,* LAMP* (loop-mediated isothermal amplification) Loopamp* Leishmania* Detection Kit,* DAT* direct agglutination test, *PCR* polymerase chain reaction, *RFLP* restriction fragment length polymorphism; for other abbreviations, see Table 1

DNA may be acquired from a vast range of clinical specimens, and each approach has varying advantages in terms of diagnostic sensitivity and specificity, ease of collection, transport and storage and invasiveness. For CL, the punch biopsy is the most commonly performed diagnostic procedure, but less invasive sampling can be achieved by using skin scrapings, fine needle aspiration and swabs, although sensitivity is sacrificed with these methods [131–134]. For post-kala-azar dermal leishmaniasis, split skin smears and skin biopsies are most commonly used [135]. In VL, splenic, bone marrow or lymph aspiration are used, and sensitivity increases with the invasive nature of the sampling method (93–99% for spleen, 53–86% for bone marrow, and 53–65% for lymph [136]). Specimens obtained from less invasive procedures, e.g., the taking of peripheral blood, are being explored for VL testing. Testing of peripheral blood by PCR is associated with a vast range of reported sensitivities, 62—93.2%, depending on the timing of sample collection during the infection process, or the fact that some *Leishmania* species may circulate at lower levels in the peripheral blood [137, 138]. The use of peripheral blood is also being explored as a diagnostic option for CL, and a limit of detection of 0.1 parasites per reaction for *Leishmania *(*Viannia*) spp. parasites has been achieved [139]. Optimal sampling, storage and transport to a receiving laboratory (such as the Centres for Disease Control and Prevention, US, or Fiocruz, Brazil) is critical. Before detection, DNA must first be extracted and purified; this is often performed by using commercial kits, such as the silica-based DNeasy Blood and Tissue Kit (Qiagen, Germany), NucliSENS easyMAG system (BioMerieux, France) or via in-house extraction protocols based on phenol extraction and ethanol precipitation [36, 140, 141]. Some rapid extraction methods have been developed recently, e.g., SpeedXtract (Qiagen, Hilden, Germany), which greatly reduce the time to test result [142]. These methods may be performed manually or through the use of automated systems, as discussed below.

Standardisation of protocols and quality control for assays that are used to detect *Leishmania* are important, particularly for molecular techniques [143]. Only few studies have compared sampling, extraction, gene target choice and primer design, and some of the findings differ from one report to an other [55]. These inconsistencies can be limited if experiments incorporate controls, which is of particular importance in settings where re-testing is expensive or the number of specimens limited. Poor DNA recovery due to losses during extraction and degradation during storage was determined by the addition of both an external and internal control to a conventional PCR (cPCR) assay for *Leishmania* [143]; DNA recovery was poor for 15.1% of samples, and a reliable result was not produced for up to 1/6 of the samples [143]. Furthermore, few multi-site studies have been undertaken to validate protocols and examine their reproducibility. An endogenous extraction control used in the measurement of a host sequence can help to account for sample quality and extraction efficacy—two major issues associated with PCR methods—and may also be used to normalise parasite load [122, 143]. For this, an exogenous internal control is spiked into a sample at a known concentration, and sample inhibition is indicated if the control is not detected or is detected at lower levels than expected. This is especially useful for potential inhibitors in samples, such as in peripheral blood; a human β-actin gene was used in a real-time PCR assay to control for this [144]. Other controls that may be included are external positive controls that are used to assess the performance of the PCR, negative template control for PCR contamination, or a negative process control for contamination of the extraction process. Laboratories should also enrol in some form of an external quality control testing program, such as the Pan American Health Organization’s Regional External Quality Assessment Program (developed for microscopic diagnosis), as an indicator of performance [55, 145].

A molecular technique that can be easily used in a resource-limited setting is nucleic acid sequence-based amplification (NASBA), which is based on the isothermal amplification of nucleic acids by enzymatic action. However, NASBA is prone to contamination, as it is not a closed tube system, which potentially leads to false–positive results. It can be used as a quantitative test, targeting RNA in the DNA background, with one method targeting 18S rDNA showing a sensitivity of 79.8% and specificity of 100% [146]. Quantitative NASBA can be combined with electrochemiluminescence, although this is more expensive and time-consuming; however, as the reaction includes a fluorescent beacon, it may be used in a real-time, closed tube format [147]. Loop-mediated isothermal amplification is another alternative to PCR that may be used in an endemic setting, as only basic equipment is required, with no need for a thermal cycler, and a total amplification time of about 40 min [148]. It involves amplification in a water bath and a visible colour change, which can be detected by the naked eye or under blue light, and more recently, in real-time by fluorimetry [149–151]. Sensitivity of this technique has been reported as 80–90% with specificities of 94–100% for human *Leishmania* diagnosis [152]. Additionally, as it is a closed tube test there is no need for post-amplification handling, thus the risk of laboratory contamination is low [153]. Recombinase polymerase amplification (RPA) and recombinase-aided amplification (RAA) are isothermal amplifications wherein recombinase enzymes and proteins avoid the need for temperature cycling as used in PCR methods [154, 155]. In RPA, the recombinase is derived from a phage, whereas in RAA, it is derived from bacteria and/or fungi. RPA was paired with a rapid extraction method to detect *L. donovani* in two studies [142, 156]; the resultant detection systems were rapid, mobile and avoided the need for refrigerated reagents. The kDNA minicircle target was used in both assays, which had sensitivities and specificities of 100% and 100% [142] and 65.5% and 100% [156], respectively. However, there are challenges in the use of RPA and RAA, as robust design guidelines have yet to be published for either method, and both are time-consuming, labour intensive and expensive.

#### Polymerase chain reaction-based detection methods

The use of polymerase chain reaction (PCR) as a molecular technique for *Leishmania* detection, in which purified nucleic acids of the pathogen are amplified, is becoming more widespread. A PCR product may be detected at the end of amplification (cPCR) by gel electrophoresis, amplified further before detection (nested PCR) or detected as amplification occurs (real-time PCR) [157–159]. PCR has the best-reported sensitivities and specificities of all the diagnostic methods, and has been suggested as the future gold standard for *Leishmania* detection [123, 132, 160, 161]. One systematic review and meta-analysis reported sensitivities of up to 100% and specificities up to 100% [136]. Another systematic review assessing assays designed for New World parasitic species regularly found limits of detection of less than one copy [119]. Several commercially available kits based on PCR amplification and detection of *Leishmania* genes have been developed and are given in Table 5. PCR performance depends on certain factors, such as the nucleic acid extraction method employed, the type of sample, the copy number of the gene target and the design of the primers that target these [53, 109]. Thus, in-house PCR methods, which are developed and used widely, have great differences in the types of DNA targets used, primer and probe design and PCR cycling protocol [129]. Technology transfer from the research and development phase does not always occur, and as these types of PCR assays are far from standardised, data comparison between laboratories is compromised [109, 162]. More recently, PCR has been used to monitor a subject of growing concern in *Leishmania* infections, particularly in endemic regions: the relapse of disease or resistance to chemotherapy. Both issues present major challenges for the control of leishmaniasis [163]. A PCR-based study that monitored parasite load in VL showed that the presence of 10 parasites/mL of blood after treatment indicated relapse, thus gave useful information for disease prognosis [164]. Four single nucleotide polymorphisms of cysteine protease B gene were identified and indicated resistance to a widely used drug, amphotericin-B; thus, detection of these could be incorporated into a PCR assay [165]. These techniques have yet to be standardised, thus are not widely available in clinical settings, but have great potential to aid public health responses [166].

**Table 5:** Commercially available DNA-based diagnostic kits for detection of *Leishmania*

Product	Supplier	Technology	Gene	Reported sensitivity	Species detected	Regulatory approval (agency)
EasyScreen *Leishmania* Detection Kit	Genetic Signatures	qPCR	18S rRNA	10 Copies/PCR	Pan-*Leishmania*	No
Genesig*Leishmania* (all species)	Primer Design	qPCR	*cytb*	100 Copies/PCR	Pan-*Leishmania*	No
*Leishmania infantum* and *Leishmania donovani* PCR Kit	MyBioSource	qPCR	DNA pol I protein B	100 Copies/PCR	*L. infantum* and *L. donovani*	No
*Leishmania* OligoC-TesT	Coris BioConcept	NASBA-OC	18S ribosomal	1 Parasite/PCR	Pan-*Leishmania*	No
*Leishmania major* PCR Kit	MyBioSource	qPCR	ND1	100 Copies/PCR	*L. major*	No
*Leishmania* sp. PCR Detection Kit	BioKits	cPCR	NR	20 Copies/mL	Pan-*Leishmania*	No
*Leishmania tropica* PCR Kit	MyBioSource	qPCR	GP63	100 Copies/PCR	*L. tropica*	No
Loopamp *Leishmania* Detection Kit	Eiken	LAMP	18S rRNA/kDNA minicircle	NR	Pan-*Leishmania*	No
SMART Leish^a^	Cepheid/WRAIR	qPCR	16S rRNA/GPI gene	4 Copies/PCR	CL causative species	Yes (FDA)
STAT-NAT *Leishmania* spp.	Sentinel Diagnostics	qPCR	NR	NR	Pan-*Leishmania*	No

*NR* Not recorded,* FDA* Food and Drug Administration (USA),* qPCR* real-time PCR,* NASBA-OC* nucleic acid sequence-based amplification-oligochromatography,* WRAIR* Walter Reed Army Institute of Research, *cPCR* conventional PCR,* kDNA* kinetoplast DNA; for other abbreviations, see Table 1

^a^The SMART Leish Kit is intended for use only in US Department of Defense laboratories

cPCR: In this method, DNA is amplified using a thermal cycler, amplicons are separated by electrophoresis due to their molecular weight and detected by staining (usually ethidium bromide) and ultraviolet light (via a transilluminator) [167, 168]. This method can be time consuming, requires an array of equipment and, as the PCR tube containing amplicons must be opened for electrophoresis, is associated with a risk of contamination [169]. Clinical sensitivity and specificity of up to 100% each have been achieved for lesion samples, with detection of as low as 0.01–0.1 pg of cultured *Leishmania* promastigote DNA [170].

Nested PCR: This method is used to overcome poor sensitivity and specificity [171, 172]. Two sequential PCRs are used: first, an outer set of primers is used to amplify the target gene (first round), then the amplicons of this PCR are re-amplified with a set of inner primers (second round) [173]. The disadvantages of this approach are that the use of two PCRs is more time consuming and requires more reagent, and since the amplicon tube is opened for the second PCR, it is an open system, which poses a contamination risk [174]. Encouragingly, however, 100% sensitivity and 100% specificity were reported for a recently developed Leishmania spp.-specific modified version of a nested PCR that was created to reduce carryover and cross-contamination [175].

Real-time PCR: This method, in which fluorescent dyes give a visual indication of amplification as the reaction occurs, is a more recent advance than cPCR [122, 176]. Although limiting in some settings due to the equipment and expertise necessary, real-time PCR is faster than cPCR and is a closed system, so contamination risk is reduced [147, 177]. Real-time PCR is generally used with intercalating dyes, e.g. SYBR green due to its lower cost, but this method is prone to false–positives as it will visualise any amplified double-stranded DNA [178]. Use of probe-based real-time PCR increases specificity, thus avoids this issue, and targets can be multiplexed, whereby multiple assays occur in the same reaction, which saves reagents and time and increases throughput [179]. Real-time PCR was compared to cPCR, and higher sensitivity and specificity, 93.9% and 100%, respectively, for the former was reported compared to two cPCR assays tested (75.6% and 100% for kDNA, and 53.7% and 88.8% for ITS1, respectively) [180]. Higher sensitivity, specificity and reproducibility of real-time PCR have also been reported in other studies [181–184]. For example, using peripheral buffy coat from cases of VL, 100% sensitivity and 100% specificity were achieved with real-time PCR [103, 129, 185]. The technique can give data on parasite load, which in turn provides information for prognosis and treatment, and is useful for epidemiological studies [186, 187]. Bisulphite modification is a method for reducing the complexity of the genome prior to application of PCR that has been adapted to *Leishmania* detection in real-time PCR [124, 188]. This assay achieved an analytical sensitivity of 10 genomic copies per real-time PCR reaction, and clinical sensitivity of 97.0% and specificity of 100.0%. Through treatment with sodium bisulphite, cytosine is converted to uracil and ultimately thymine during the first round of PCR. This causes the genomes of subtypes to become more similar to each other, making genus-level primer and probe design possible for highly polymorphic gene targets [189]. The resulting simplified genome enables the design of simplified primers with unique characteristics, and the consequently fewer mismatches result in a similar melting temperature (Tm), allowing for the design of longer oligonucleotides, thereby increasing the specificity of the assay. Additionally, proximate Tms between species-level oligonucleotides can be achieved, making multiplexing more efficient, as a uniform PCR cycling protocol can be easily designated [124].

Droplet digital PCR: The advent of this method has enabled the absolute quantitative measurement of target DNA, negating the need for calibration curves in PCR assays [190]. DNA molecules are partitioned into tens of thousands of replicate PCR reactions, and amplification to endpoint PCR occurs, at which point each “droplet” has template or no template present [191]. Due to the vast numbers of binary (positive or negative) results, the number of target DNA molecules can be calculated precisely. A droplet digital PCR was developed based on 18S rDNA and validated for seven *Leishmania* species; however, despite the accurate quantification of DNA, the assay was marginally less sensitive and specific than an equivalent real-time PCR assay (84.0% for the former vs 85.0% for the latter) [192]. Moreover, the authors reported that the cost of droplet digital PCR is three times that of real-time PCR, thus is not suitable for the routine diagnosis of *Leishmania*.

## Methods for the identification of species of *Leishmania*

Differentiation of *Leishmania* species to discriminate the species, or group of species, that cause disease is important, both clinically and epidemiologically, for disease prognosis, determining therapeutic options and surveillance of populations [122, 193]. As in *Leishmania* detection, no single differentiation method is considered to be a gold standard, although several techniques have been proposed, including PCR, multilocus enzyme electrophoresis (MLEE) and multilocus sequence typing (MLST) [122, 133, 193–198]. The lack of a definitive gold standard for species typing may be attributed to inherent issues related to a lack of standardisation, particularly with regard to the interpretation of the typed result. This pitfall is complicated by the closely related *Leishmania* species and inter-species hybrids that exist, and the novel species that continue to be discovered [199, 200]. Moreover, methods of interpretation differ between laboratories and, if species assignment is based on centralised programs like BLAST, results can be dependent upon the evaluation of resultant similarity scores [201]. Despite this, multiple robust methods exist and the results obtained with their use continue to strengthen the molecular epidemiological, taxonomic and clinical databases, even if these methods are not yet optimal for routine uses.

Multilocus studies are now used in preference to single locus analysis for population-wide studies as they achieve better resolution [129]. By combining genomic targets in parallel, multilocus schemes capture genetic relationships that may be missed by one genetic locus, which is particularly advantageous in intraspecific variation studies or for species typing within species complexes due to the amount of information gathered [6]. MLEE distinguishes between organisms through electrophoresis of enzymes, and is regarded by some as the gold standard for *Leishmania* typing and taxonomic studies; information acquired using MLEE led to the development of the first phylogenetic trees of *Leishmania* [18, 202, 203]. Differences in enzyme mobilities are due to differences in their protein structures, which comprise different amino acids, and lead to the creation of banding patterns from which zymodemes (populations with similar isoenzyme patterns) can be assigned [24]. MLEE is laborious as it requires a large volume of cell culture and can take 1–2 months to produce results, which cannot be compared, with confidence, between laboratories; e.g., different enzyme panels are used in Europe and South America [204, 205]. Furthermore, one zymodeme, MON-1, for *L. infantum*, the causative agent of most cases of VL in the Mediterranean Basin and South America, was shown to be heterogeneous and polymorphic [129].

### DNA-based identification methods

MLST, which involves DNA sequencing sections of defined housekeeping genes (usually seven or more) and the many allelic combinations produced, results in unambiguous characterisation of isolates, giving both inter- and intra-species information on heterogeneity [206, 207]. This method is considered so powerful it has also been proposed as the new gold standard for taxonomic determination of *Leishmania* [208]. MLST has high reproducibility and can be compared between laboratories; however, it is technically demanding [209].

Like MLST, multilocus microsatellite typing (MLMT) uses co-dominant markers, and because of the relatively high mutation rate of microsatellites, comparison of closely related organisms is possible [209, 210]. It works by the amplification of repeat sequences found in microsatellites, where polymorphisms in the copy number of repeats define the type assigned [211]. For instance, MLMT has been used to analyse *L. donovani* strains. In one study, the identification of heterogeneous genotypes by MLMT negated the usefulness of MLEE determining genetic relationships in zymodeme MON-37. Not only were the isolates genetically diverse but, geographically, they were spread globally, leading the authors to surmise that the discriminatory power of MLMT adds depth to both diagnostic and population genetic studies [212].

DNA sequencing is based on the classic Sanger sequencing (chain termination) method and, more recently, next generation sequencing (NGS), both of which identify the precise order of nucleotide bases in a targeted DNA locus [213]. DNA sequencing provides important information for genetic, clinical and epidemiological studies. For instance, gene sequence analysis has been used to detect *Leishmania* hybrids using the cytochrome b gene, to study differences in genetic composition between healed and non-healed patients using the ITS1, 7SL RNA and heat-shock protein 70 regions, and to illuminate the diversity of kDNA minicircle classes [213–215]. It is viewed currently as an impractical technique for endemic areas, as it is technically demanding, although simplified techniques such as nanopore sequencing are being developed [198, 216].

NGS involves the extremely high-throughput sequencing of nucleotides in parallel [217]. It provides deep genome sequencing and whole genome sequencing (WGS) of organisms, and provides population-level data in a clinical context, although it is too costly for routine typing [218, 219]. The first complete genome sequence of any *Leishmania* parasite (i.e., *L. major*) was completed in 2005 and, since then, the complete genomes of *L. mexicana, L. tropica*, *L. amazonensis*, *L. donovani*, *L. infantum*, *L. panamensis*, *L. braziliensis*, *L. guyanensis*, *L. naiffi*, *L. peruviana*, *L. lainsoni, L. martiniquensis* and *L. orientalis* have been sequenced [220, 221]. One type of NGS couples WGS with MLST, and is a methodological advance for the provision of standardised epidemiological, molecular evolution and pathogenicity data [222]. Aneuploidy, which is observed in laboratory cultivated samples, as well as mosaicism, can be challenging in WGS, and thus read depth is critical for correct interpretation of *Leishmania* NGS data [223–225].

#### PCR-based identification methods

Restriction fragment length polymorphism (RFLP) technology: This methodology is used after amplification by PCR, and is widely applied in species identification [199]. Post-PCR amplicons are digested with a restriction enzyme and the products are detected on a gel. The banding pattern produced can be used to identify a particular species, depending on the presence or absence of a restriction enzyme site in the PCR product derived from the target [226]. It is a relatively inexpensive technique compared to real-time PCR, and a multitude of gene targets can be utilised, including heat-shock protein 70, glycoprotein 63, kDNA, cysteine protease B, mini-exon or NADH dehydrogenase subunit 7 [121, 227–231].

**Fig. 3:**
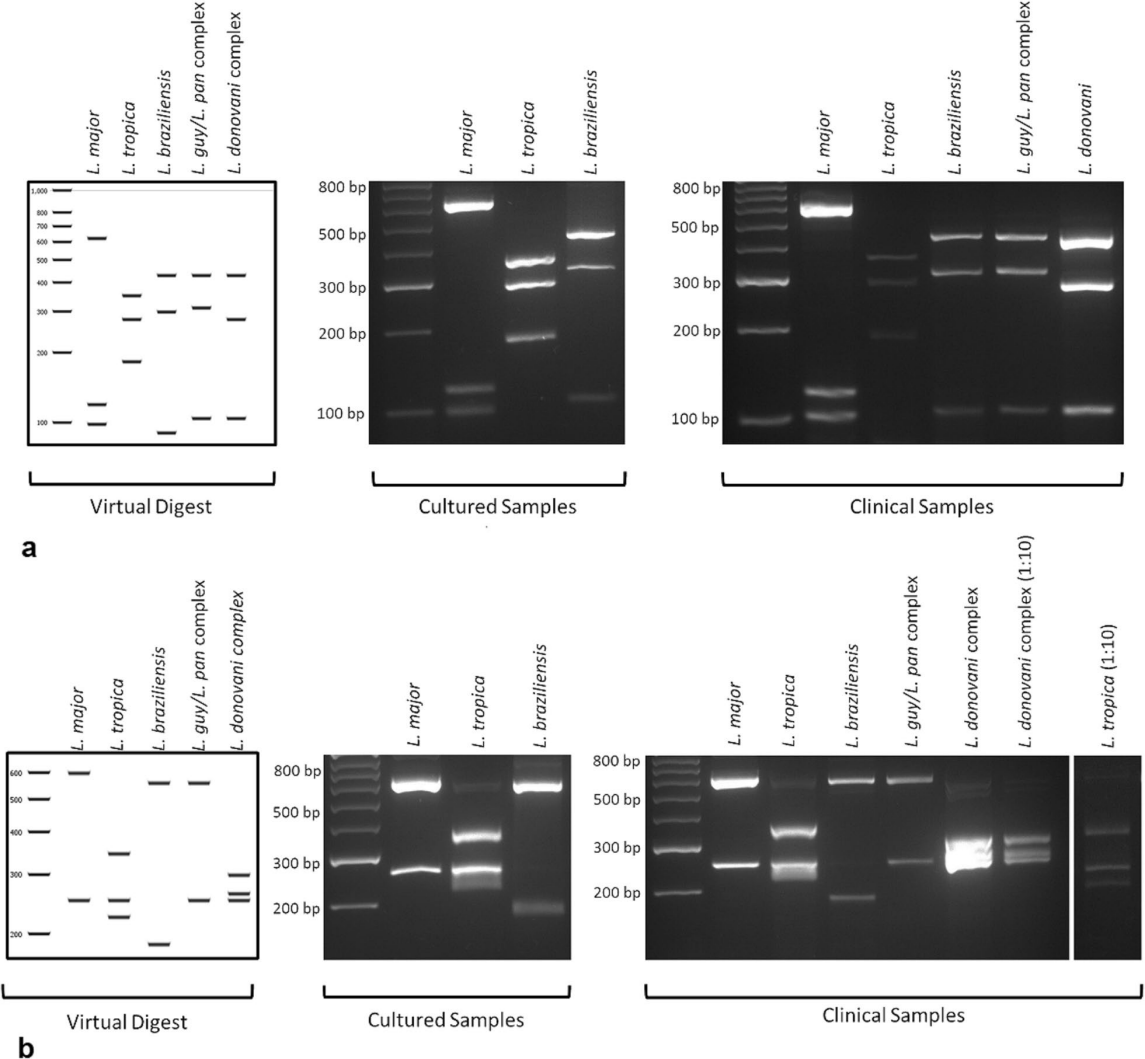
Polymerase chain reaction—restriction fragment length polymorphism analysis of *Leishmania* species targeting the NADH dehydrogenase subunit 7 gene, digested with *NIa*III (**a**) or *Hpy*CH4IV (**b**) run alongside 50-base pair (*bp*) molecular weight markers. Digestion of in silico, culture and direct clinical samples enabled differentiation of *Leishmania* species. Reprinted with permission of Kaufer et al. [231]

An ITS1-RFLP reported by Schönian et al. [231] was able to differentiate most *Leishmania* species but was less useful for those species within the *L. braziliensis* complex [232, 233]. Such findings can be problematic, as the clinical presentation of the MCL tropism is caused by more than one of these species, but the response to treatment differs between them [129]. Improvements in species discrimination using RFLP were achieved recently using the NADH dehydrogenase subunit 7 maxicircle gene target, where it was possible to discriminate *L. braziliensis* from other species within the *L. braziliensis* complex (Fig. 3) [231]. RFLP presents multiple difficulties, as it needs a relatively high parasite load, and is thus often paired with cell culture, and multiple restriction enzymes may have to be employed depending on the DNA target. Additionally, RFLP can be difficult to compare between laboratories as banding patterns may be dissimilar due to differing gel size or concentration [234].

Melt curve analysis: This method is used to differentiate species following real-time PCR. Its effectiveness relies on the fact that the temperature at which a sequence of double-stranded DNA dissociates (or “melts”) is a function of the GC/AT ratio and the length of an amplicon [235]. Different species exhibit different melting points, which allows for discrimination [236]. In a study that used Tms to group infecting species, species that caused different clinical presentations (i.e., CL/MCL and diffuse cutaneous leishmaniasis) could be differentiated [234]. Either a standard melt curve or high resolution melt can be use for analysis, the latter being a method that is able to detect more subtle differences in temperature, which potentially gives better species discrimination [237–239].

### Biosensors

Biosensors lead the emerging field of nanodiagnostics, spanning target detection of DNA/RNA, proteins and even volatile organic compounds from exhaled breath. They are devices that, put simply, convert a biological signal into an electrical signal via a recognition element; they are reported to be low-cost and portable, with high sensitivity and specificity of performance documented [240, 241]. Biosensors use one of several modes of signal generation, like electrochemiluminescence or optical signals, or are based on surface plasmon resonance (SPR). Genosensors, recognising DNA (or RNA), dominate biosensor diagnostics for *Leishmania.* Other recognition elements may be antibodies, antigens or the newer aptamer-based sensors [242]. Aptamers show great promise in binding biological targets with high affinity; they are short, single-stranded nucleic acids that form unique three-dimensional structures and recognise and bind target molecules in a similar fashion to antibodies, such as those targeting the *L. infantum* histone H3 or poly(A) binding protein [243, 244]. A DNA-based biosensor using SPR that targeted the kDNA detected *L. major* and *L. tropica* [245]. Also using SPR biosensing techniques, Ferreira et al. [245] developed an immunosensor based on circulating antibodies against *L. infantum*, which achieved antibody detection within 7 min [246]. Another DNA detection biosensor, which uses fluorescent probes based on the kDNA of *L. infantum* and nanostructured films as sensing platforms, provided sensitive results (1.1 nM of target DNA) even for complex sample types such a human blood [247]. To determine selectivity for the target molecule, the authors measured fluorescence recovery intensity when a target DNA sequence with a single base mismatch was introduced, and observed a reduction of 32% when compared to a fully complementary sequence [247]. Most recently, another genosensor based on the recognition of a single-stranded DNA sequence of *L. infantum* on cadmium sulfide nanosheets was described; the detection limit for *L. infantum* DNA was 1.2 ng/uL without reaction with *L. major* and *L. tropica* DNA [248]. Biosensor development for *Leishmania* detection is in its early stages and requires more research to improve efficiencies and standardisation. Despite this, recent publications on their use in NTDs have indicated the potential for their increased performance as well as a reduction in interactions with interfering substances, good stability and the miniaturisation of devices, allowing their portability [249]. Furthermore, this detection method has been widely integrated into smartphone technology, simplifying the interpretation of results and allowing for multiplexing of targets, such as multiple species [250, 251]. Biosensor technology, though in its infancy with regard to leishmaniasis detection, may be a good solution to the challenge of providing a cost-effective, fast and portable detection method [252].

## Conclusions

**Fig. 4:**
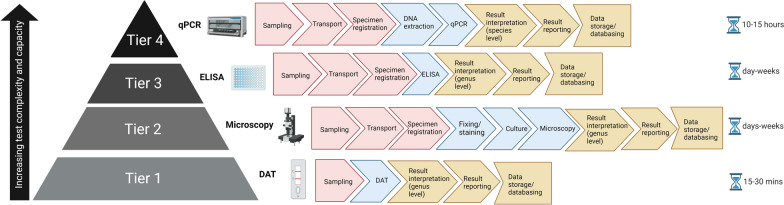
Example workflows for the detection of *Leishmania* in clinical diagnostic laboratories at each laboratory tier, as set out in the list of essential in vitro diagnostics (World Health Organization) [253].* qPCR* Real-time PCR,* ELISA* enzyme-linked immunosorbent assay, *DAT* direct agglutination test

A multitude of diagnostic assays exist for the detection of *Leishmania* species, but there is no widely accepted gold standard [122, 195, 196]. There have been, however, huge developments in the speed and accuracy of methods with advances in technology, and different approaches may be better suited to different diagnostic health care settings, ranging from primary health care centres (where technical staff perform point-of-care or single-use tests for outpatients) to district hospital laboratories (where limited numbers of staff perform selected routine tests), regional or provincial hospital laboratories (where high numbers of laboratory staff are present to cover many pathology disciplines) and, ultimately, to national reference laboratories (providing highly specialised tests, education and training in research or for teaching hospitals) [253]. All the techniques for the detection and identification of *Leishmania*, of which there is a vast array, have their own strengths and limitations, but high sensitivity, high specificity, low turnaround times and affordability are the critical features of an ideal test. Also important is discrimination between *Leishmania* species, which is vital for epidemiological studies, disease prognosis and for the implementation of patient treatment regimens. Figure 4 illustrates typical workflows for the techniques used in diagnostic laboratories, and highlights that, whilst more traditional protocols can be used to identify *Leishmania* to the genus level relatively speedily when in the hands of trained, experienced professionals, only molecular-based techniques can give species-specific diagnostic information. As global efforts increase to control and eliminate NTDs, there is a need to develop, validate and standardise novel diagnostics for the detection and differentiation of *Leishmania* spp. With the successful implementation of such methods, the global burden of this disease could be reduced dramatically, with positive outcomes being seen for the people that need them the most.

## Data Availability

Not applicable.

## Abbreviations

CL: Cutaneous leishmaniasis
cPCR: Conventional polymerase chain reaction
ELISA: Enzyme-linked immunosorbent assay
ICT: Immunochromatography test
MCL: Mucocutaneous leishmaniasis
MLEE: Multilocus enzyme electrophoresis
MLMT: Multilocus microsatellite typing
MLST: Multilocus sequence typing
NASBA: Nucleic acid sequence-based amplification
NGS: Next-generation sequencing
NTD: Neglected tropical disease
PCR: Polymerase chain reaction
RAA: Recombinase-aided amplification
RFLP: Restriction fragment length polymorphism
RPA: Recombinase polymerase amplification
SPR: Surface plasmon resonance
Tm: Melting temperature
VL: Visceral leishmaniasis
WGS: Whole genome sequencing
WHO: World Health Organization
